# Classification of Chest X‐Ray Images Using Modern Machine Learning Techniques by Orang3 Data Mining Tool

**DOI:** 10.1155/ijbi/8859105

**Published:** 2026-10-06

**Authors:** Amani Al-Ghraibah, Areen Arabiat, Muneera Altayeb

**Affiliations:** ^1^ Biomedical Engineering Department Jordan University of Science and Technology Irbid Jordan just.edu.jo; ^2^ Communications and Computer Engineering Department Al-Ahliyya Amman University Amman Jordan ammanu.edu.jo

**Keywords:** adaptive boosting, chest diseases, gradient boosting, machine learning, Orange3 data mining tool, random forest

## Abstract

During the COVID‐19 pandemic, there were many concerns raised in diagnosing chest diseases. Regular diagnosis processes of chest diseases sometimes fail in distinguishing between Corona, viral pneumonia diseases, and normal chest x‐rays through regular assessments which also need time and not as accurate as the automated diagnosis based on machine learning. Chest diseases can be sometimes diagnosed using x‐ray images where these images are used to identify if the patient is normal or has one of the chest diseases like Corona and viral pneumonia. Modern machine learning techniques have achieved high performance in aiding medical diagnostic processes. The novelty of this work lies in a wavelet‐based feature extraction framework coupled with a systematic evaluation of ensemble learning algorithms for accurate and computationally efficient classification of COVID‐19, viral pneumonia, and normal chest x‐ray images. The number of patients used in each case is 1200 patients and this huge dataset is used to create classification models using adaptive boosting (AdaBoost), gradient boosting (GB), and random forest (RF) machine learning algorithms using Orange3 data mining tool. Using wavelet features, the results of evaluating the proposed models were 99.5% on average. The proposed models are very effective in identifying chest diseases and can be combined with x‐ray machines in hospitals, so physicians can determine the best treatment for patients. The proposed methods beat other researchers found in literature as they are faster and more accurate where each step takes few seconds on average to complete.

## 1. Introduction

After the COVID‐19 pandemic, chest diseases have become increasingly prominent in public health discussions. COVID‐19, caused by the SARS‐CoV‐2 virus, primarily affects the respiratory system and can cause other complications. Symptoms range from mild respiratory matters to severe respiratory failure, making it a serious global health concern since its appearance in late 2019. On the other hand, various viruses including influenza, respiratory syncytial virus (RSV), and coronaviruses other than SARS‐CoV‐2 can cause viral pneumonia. It typically exhibits symptoms like fever, cough, and difficulty in breathing. Viral pneumonia, although typically less severe than bacterial pneumonia, can still cause significant illness, especially in vulnerable populations like the elderly and individuals with underlying health conditions.

Both diseases underscore the importance of comprehending respiratory infections, their transmission, prevention strategies, and the influence of vaccines. It is essential to address these respiratory conditions to enhance respiratory health and decrease mortality rates linked to viral infections ([1, 2]). Given the global expansion in the chest disease′s spread and the high casualty rate across all nations, it is critical to develop fast and accurate disease identification techniques. The RT‐PCR was the primary method for diagnosing COVID‐19, whereas viral pneumonia was diagnosed using specific blood tests to confirm the infection and to identify the germ that is causing the illness. In addition, a chest x‐ray is used to look for the location and extent of inflammation in the lungs. In reality, the regular laborious manual methods take a long time and can have high diagnosis errors. Concerns are further raised by the shortage and difficulty of RT‐PCR test kits, which pose a risk to the quantity of COVID‐19 cases. These days, a quick and accurate method for identifying and classifying chest diseases is required. Therefore, when compared with the RT‐PCR technique, radiography is an effective tool to distinguish between different chest diseases like coronavirus and viral pneumonia [1, 3].

The clinical workflow of computed tomography and chest radiography has steadily been impacted by the use of computer‐assisted detection (CAD) for chest diseases. The 1960s saw the first attempt to create a CAD system, and research has indicated that using an x‐ray CAD system as a helper improves the accuracy of chest illness detection The majority of CAD systems currently in use are intended to detect lung cancer early on, but because of the intricacy of chest x‐rays (CXRs), the automatic diagnosis of diseases is still unsolved. The automatic detection of different forms of diseases is the subject of comparatively few investigations [4].

There are many notable works in the literature that have utilized machine learning techniques for chest disease detection from x‐ray images. A deep transfer learning‐based model is used in Chhikara et al. [5] work for the automatic detection of COVID‐19 from CXRs using the Inception‐v3 model, in which they applied flattening, node dropping, normalization, and dense layer. The proposed method is based on deep neural networks for feature extraction and classification of the images. They tested the effectiveness of the designed model on three radiography datasets including three different classes: COVID, normal, and viral pneumonia. The proposed model reached an accuracy of 97.7%, 84.95%, and 97.03% on each of the three datasets.

A model for COVID prediction from CXRs using CheXNet is presented in Haritha et al. paper [6]. The presented model classifies CXR images into two classes (COVID and normal) using CheXNet which is basically a convolutional neural network (CNN) model that used ChestXray14 dataset, and it is trained to detect abnormalities in CXRs. The model classified images with 99.9% accuracy. A deep CNN model with transfer learning was proposed in Chhikara et al. [7] work. Before building the model, a preprocessing step is done using filtering, gamma correction, equalization, and compression methods. The proposed model is done on standard x‐ray dataset collected from a private medical center, and it is compared with ResNet, ImageNet, Xception, and Inception using the common metrics like precision, recall, accuracy, and ROC accuracy score. Their results reveal the results of other conventional models found in literature.

In Hashmi et al. [8] paper introduced a novel approach based on a weighted classifier which combines the weighted predictions from many deep learning models such as ResNet18, Xception, InceptionV3, DenseNet121, and MobileNetV3 together in a good way. Basically, a network is used to predict the classes based on the quality of the provided dataset using a supervised learning method. Also, transfer learning was used to modify the deep learning models to get higher training and validation accuracy. At the end, data augmentation techniques are employed partially to increase the training dataset in a balanced way. The presented combined weighted classifier model is able to outperform most of the individual models.

Jaiswal and his colleagues [9] identified a deep learning‐based approach to detect the localization of pneumonia in CXRs images. They applied a critical modification in the training process to achieve robustness and a novel postprocessing step that merges bounding boxes from multiple models. Their work presents an identification model that achieves better performance evaluated on chest radiograph dataset which depicts potential pneumonia causes. Hwang et al. [10] used a commercialized deep learning‐based computer‐aided detection (CAD) system for evaluating the CXRs. The CAD was designed to detect pulmonary nodules or masses, pulmonary infiltrates, and pneumothoraxes on CXRs. Their results recommended that the CAD system can be a supportive part for physicians in interpreting CXRs and serving image‐based classification of COVID‐19 patients in resource‐constrained environments.

Lenny et al. [11] employed pretrained CNN models as feature extractors in combination with supervised classifiers to distinguish between normal and pneumonia‐affected x‐rays. Their results demonstrated high classification accuracy (CA), highlighting the effectiveness of transfer learning in medical image analysis. Nevertheless, the method depends heavily on pretrained models derived from nonmedical image datasets, which may limit generalization across diverse clinical cases, and it did not fully address challenges such as data imbalance or the feasibility of real‐time application in clinical settings. Owida et al. [12] paper presented a new diagnostic tool for investigating COVID‐19 disease using advanced deep learning techniques. Important features were extracted and used in the classification process. The results showed that the accuracy was 98.8% when using wavelet features, whereas the classification using mel frequency cepstral coefficients (MFCCs) features got an accuracy around 97%.

The innovation of Al‐Ghraibah et al. work [1] lies in using new diagnostic techniques to differentiate between COVID‐19 and viral pneumonia diseases using advanced AI technologies. The first step in their work was extracting some novel features from CXR images using three image processing techniques: wavelet analysis, scale invariant feature transformation (SIFT), and the MFCC. Then two machine learning algorithms were used to classify x‐ray images into two classes: the support vector machine (SVM) and the artificial neural network (ANN). The results of the experiments show that the classification using SIFT features had an accuracy of around 99%, and using wavelet features reached 98.9% which are extremely good and superior to other classification results that utilized in this research and to other studies discovered in literature.

The authors Kukker and Sharma [13] proposed a reinforcement learning‐based framework using a modified fuzzy Q‐learning (MFQL) algorithm combined with wavelet‐based preprocessing to classify CXR images for pneumonia and tuberculosis diagnosis. The model was designed as a self‐learning classifier and outperformed SVM and k‐Nearest Neighbor (k‐NN) classifiers, demonstrating high CA for disease detection and severity assessment. Also, Singh and his colleagues [14] proposed a pneumonia detection framework based on CXR images using wavelet transform and gray‐level co‐occurrence matrix (GLCM) texture features. Six time‐frequency features were extracted and classified using SVM and k‐NN classifiers. The cubic SVM achieved the best performance with an accuracy of 94.6%, outperforming the weighted k‐NN classifier, which achieved 92.6% accuracy.

These previous works illustrate the varied approaches and growing interest in applying machine learning to chest disease detection, highlighting advancements in both traditional and deep learning methodologies. In this work, wavelet analysis is used to extract five features from CXR images in the dataset which includes three types of CXR images: images with Corona disease, images with viral pneumonia disease, and normal images. Then three classification techniques through applied modern data mining tool are used to distinguish between the normal and diseased images. The methods are adaptive boosting (AdaBoost), gradient boosting (GB), and random forest (RF) machine learning algorithms using Orang3 software.

## 2. Methodology

### 2.1. Data Set

A total of 3600 gray‐scale CXR images are used in each classification process, which include 1200 cases for patients with COVID‐19 disease, 1200 cases for patients with viral pneumonia disease, and 1200 normal cases where the patient has no disease. The data are collected from online sources that have developed the database of COVID‐19 x‐ray images, and they are available on “Kaggle” website which is a free source of many types of data [15, 16]. The dataset was carefully examined to avoid duplicate samples and possible overlap between training and testing images. Data splitting was performed before feature extraction to prevent information leakage. For each classifier, the dataset was partitioned into training and testing subsets according to a special protocol described in the following subsections which is specific for each classification method, and the confusion matrices presented in the Results section were generated using the mentioned method. Figure 1 shows an example of x‐ray images of each case.

**Figure 1:**
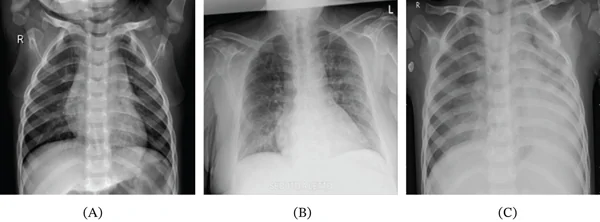
Example of CXR images of the three cases: (A) normal, (B) COVID‐19, and (C) viral pneumonia.

### 2.2. Feature Extraction

There are numerous methods used in the literature for feature extraction from medical CXR images. In this project, a new application of wavelet analysis is used to extract five features from each image in the dataset. This analysis is chosen for this study because it is well known for its ability to distinguish between various classes of the same image type. Wavelet analysis divides each image into N levels and detects an image′s details with extreme precision, which then can be used to distinguish between normal and diseased images. Following feature extraction, a variety of classification tasks is carried out using Orang3 machine learning algorithms.

Wavelet analysis is a common technique for investigating localized variations of power within a time series. Wavelets are strong tools for spatial analysis with a variety of uses in image processing. It is employed to separate the approximations and details that make up an image′s content into two distinct categories. After going through high‐pass and low‐pass filters, a picture is divided into low‐pass (approximation) and high‐frequency (details) components as shown in Figure 2. In this paper, the image is passed through five levels resulting in three detail images: horizontal, vertical, and diagonal at each level [1, 14]. The energy at each decomposition level, or what is known as the high‐pass images, is found using the three image specifics and the absolute values for the wavelet coefficients. To determine the total energy, the absolute values of the three high‐pass resultant images are then summed. Energy 1, Energy 2, Energy 3, Energy 4, and Energy 5 are the five energy features associated with each of the five decomposition levels that are recovered when the number of decomposition levels is set to five. Figure 2 shows an example of wavelet analysis in image processing.

**Figure 2:**
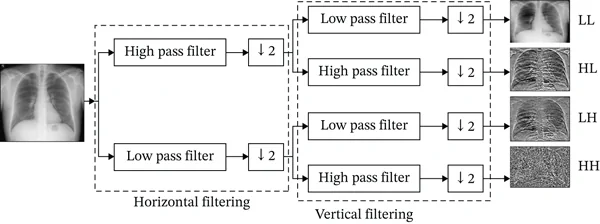
Example of the wavelet analysis in image processing [17].

Wavelet decomposition separates CXR images into multiple frequency subbands that capture both coarse anatomical structures and fine textural details. The low‐frequency (LL) subband preserves the overall lung appearance and intensity distribution, whereas the high‐frequency subbands (LH, HL, and HH) emphasize edges, local variations, and texture irregularities. Table 1 represents what each wavelet subband (LL, LH, HL, and HH represents. Pulmonary abnormalities such as ground‐glass opacities, consolidations, infiltrates, and pneumonia‐related patterns alter the normal texture and the organization of lung tissue. Consequently, wavelet‐derived texture features can quantify these changes by measuring variations in intensity distribution, spatial relationships, and structural heterogeneity within the lung fields. Previous studies have demonstrated that wavelet‐transformed texture features improve the characterization of COVID‐19 and pneumonia‐related pulmonary lesions and provide discriminative information for machine learning classification [13, 18].

**Table 1:** Interpretation of wavelet subbands in terms of image structure and texture.

Low and high frequency subbands	Image structure and texture
LL	Overall lung structure and intensity distribution.
LH	Horizontal texture variations and edges.
HL	Vertical texture variations and edges.
HH	Fine high‐frequency details and texture irregularities.

### 2.3. Machine Learning Techniques

Deep learning and machine learning are widely used to evaluate data, classify processes, and make defensible decisions based on the patterns and visions recovered from the dataset. The classification model in this work is created using a number of supervised classification techniques, such as RF, GB, and AdaBoost. The data mining Orange3 tool is used in this work, and Figure 3 represents the main interface of the software. The next sections provide an explanation of each classification method utilized in this investigation [1, 19].

**Figure 3:**
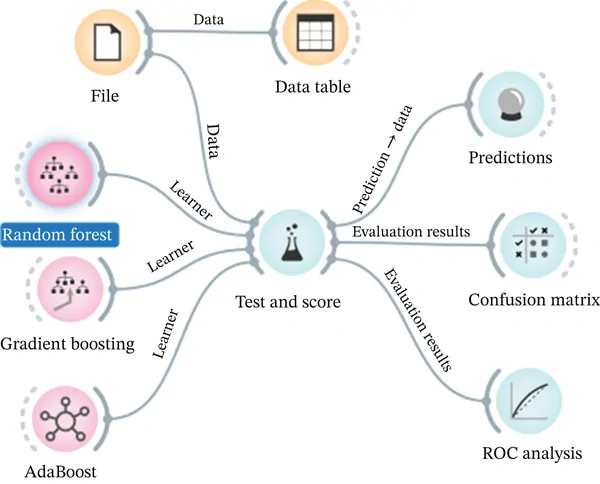
Proposed model using Orange3 data mining tool.

#### 2.3.1. AdaBoost

Robert Schapire and Yoav Freund proposed the machine learning technique AdaBoost. This technique uses many classifiers, each of which is trained by splitting the sample based on the output of the classifier that came before it. This approach enhances the adaptability and resilience to overfitting of the AdaBoost algorithm in particular classification problems. It is possible to improve the AdaBoost weak classifier by using a voting model that combines all of the weak classifiers [20]. AdaBoost is an iterative model that adjusts the weight of training samples by generating weak predictors and combining them into a strong predictor in order to enhance the model′s performance and integrate it with other learning algorithms [21]. Equation (1) shows that only the strongest tree will be added to the total, removing the generic tree in the process each time a new tree model is introduced. This way, as more calculations are performed, the overall performance of the model will gradually improve.

(1) Fnx=Fm−1x+argminhh∑i=1nLyi,Fm−1xi+hxi.

In the equation, *y*_*i*_ is the tree′s prediction result, *h*(*x*_*i*_) is the newly inserted tree, *F*_*n*_ (*x*) is the overall model, and *F*_*n*−1_ (*x*) is the overall achieved in the previous round [22]. Figure 4 depicts the AdaBoost model architecture.

**Figure 4:**
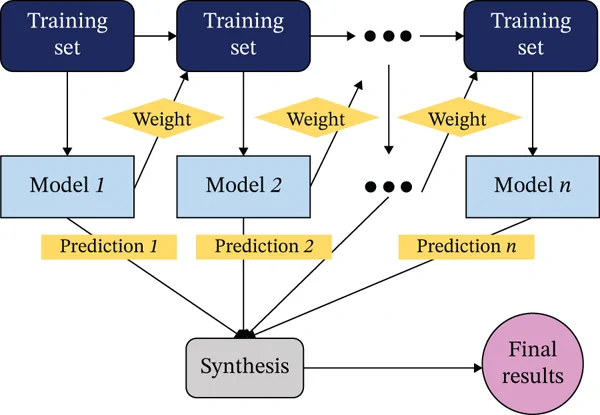
AdaBoost model architecture [22].

#### 2.3.2. GB

GB is an ensemble learning technique that unifies several models into a single final model. As the weak learners are trained one after the other, each estimator is introduced gradually by varying the weights. The GB approach predicts the residual errors of previous estimators in an effort to minimize the difference between the projected and actual values. The gradient technique is used to reduce the loss in the same way as neural networks alter weights through GB. Extreme gradient boosting (XGBoost) is a version that uses gradient enhancement to discover which model performs best by producing more accurate predictions. It computes the gradients of the loss function to reduce overfitting and enhance model performance and generalization. It is user‐friendly and performs well with big datasets [23, 24]. GB is a suitable option for multiclass task classification because it can incorporate many weak models in a way that allows them to learn from the mistakes of the previous models and raise their accuracy [25]. Since each  *f*_*i*_( *x*_*t*_) is a regression‐t decision tree, the total of n regression‐trees is known as the gradient boosting tree (GBT) *F*_*n*_(*x*_*t*_), as illustrated in Equation (2) [26].

(2) Fnxt=∑n=1nfixt

#### 2.3.3. RF

One of the more reliable methods for classification and regression is RF. Breiman created the algorithm in 2001 after Ho initially introduced it in 1995. It builds an ensemble of trees by training each one on two‐thirds of the training set and evaluating its performance using a technique known as “bagging” or bootstrap aggregating. The method ensures accuracy and stability by reducing model variance [27].

This method uses decision trees to construct an ensemble of classifiers. Every decision tree is trained using a random sample with replacement from the original data, and the trained bootstrap samples are expected to contain 37% duplicate occurrences. A different method of randomization is attribute sampling. A majority vote determines the final forecast. RF′s default configuration is parameter‐free and performs admirably. Additional tuning parameters include establishing leaf nodes, mandating a minimum sample size, and accounting for tree depth [28].

The RF algorithm is a nonparametric supervised learning technique that combines multiple weak classifiers to produce excellent accuracy and generalization performance through averaged results using voting or mean‐based approaches [29]. In the equal‐representation, RF Equation (3), where *F*_*i*_  the value is obtained from the system and *y*_*i*_ is the original value utilized for the feature and *N* is the number of features used to identify comparable accounts [30].

(3) RF=1N∑N=1NFi−yi2.

## 3. Results

In this section, the result of using the three classification methods on the Orange3 program is presented. Figure 5 shows the proposed methodology which consists of four main stages. First, feature extraction is performed using wavelet analysis to capture the most relevant information from the input data and represent it in a compact form. Second, the extracted features are organized into a feature matrix, which serves as the input dataset for the classification models. Third, machine learning models are developed using AdaBoost, RF, and GB algorithms to learn the underlying patterns within the data and perform classification. For each classifier, the dataset was partitioned into training and testing subsets according to the protocol described in the Methodology section and it is specific to each classification method, and the resulting testing set was used to generate the corresponding confusion matrix and performance metrics. Finally, the performance of the developed classifiers is evaluated and compared using appropriate evaluation metrics, enabling the identification of the most effective model for the target classification task. The confusion matrix, which is an *N* × *N* matrix where *N* represents the number of classes or outputs, serves as a central tool in this evaluation. For binary classification, a 2 × 2 confusion matrix is applicable, whereas for scenarios involving three classes of diseases, like the one discussed in this study, a 3 × 3 confusion matrix is employed. In this work, the number of classes (targets) is 3, the number of features is 5, and the total number of images is 3600. Figure 6 shows a sample of the data where the number of instances, number of features, and classes of each row are presented to clearly understand the input used in the classification processes.

**Figure 5:**
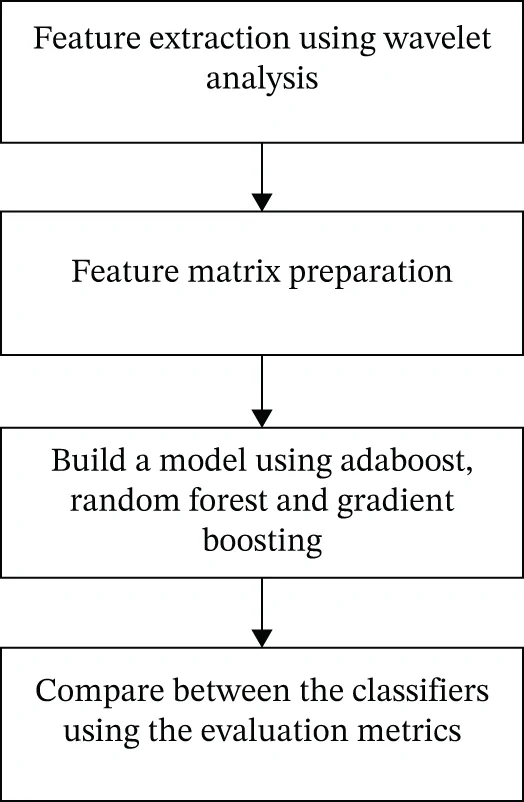
Workflow of the proposed framework.

**Figure 6:**
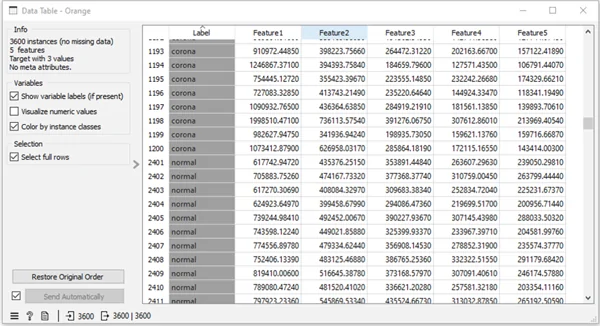
Sample of the data shows the number of instances, number of features, and classes of each row.

### 3.1. Metrics and Evaluation

There are two categories of test performance metrics: concordance metrics, which evaluate agreement between tests, and discordance metrics, which assess disagreement. In Table 2, there are two different kinds of test results: Ps and Ns which refer to concordance and discordance metrics, respectively. These metrics line up with the known concepts of test sensitivity and specificity. Every measure is defined using both conditional probabilities and the *N* × *N* table [31].

**Table 2:** Confusion matrix of three classes.

Confusion matrix		Predicted classes		
		Corona Class 1	Normal Class 2	Viral Class 3
Actual classes	Corona Class 1	P1	N1	N2
	Normal Class 2	N3	P2	N4
	Viral Class 3	N5	N6	P3

In the table, each column represents the predicted classes, and the actual classes are shown by each row. The diagonal cells of the confusion matrix represent samples that have been correctly classified, whereas the off‐diagonal cells illustrate the model′s errors in accurately predicting the true classes. P1, P2, and P3 are values that have indications about the prediction of the model that is true and correct. For example, the model predicted that P1 of the participants is healthy and has no pain out of (P1 + N1 + N2) participants. For the second row, the model predicted that P2 of the patients had moderate pain out of (N3 + P2 + N4) participants and so on. The CA of the whole predictions is calculated using Equation (4) where P1, P2, and P3 are the correct predictions.

(4) CA=P123+P+Pnumberof all patients.

All other values in the confusion matrix (N1 to N6) denote the misclassification that results from the classification process. After defining all values in the confusion matrix, now the known metrics can be calculated and defined; the first one is the true positive (TP) which is simply the P′s values in the matrix. The false negative (FN) value is derived by summing the values in each row, excluding the (TP) value, within the confusion matrix, it is shown in Equation 5 for the case of Class 0. Also, the false positive (FP) is the sum of values of the corresponding column except for the (TP) value and it is given in Equation 6 as an example of Class 0. Finally, true negative (TN) is calculated as the sum of values of all columns and rows except the values of that class that we are calculating the metrics for; it is given in Equation (7), also applied for Class 0.

(5) FN=N12+N,

(6) FP=N35+N,

(7) TN=P2346+P+N+N.

Moreover, precision and recall serve as critical performance metrics in machine learning endeavors, including object detection, pattern recognition, and classification, aiding in the extraction and interpretation of information from training datasets. The proportion of pertinent data among the recovered data is known as precision (positive anticipated) and it is presented in Equation 8. On the other hand, recall (sensitivity) refers to the percentage of pertinent facts that were successfully recovered; it is given by Equation (9) ([32]; [33]; [34]).

(8) Precision=TPTP+FP,

(9) Recall=TPTP+FN.

*F*1‐score that is specified in Equation (10) is a harmonic mean of precision and recall, and so it gives a combined idea about these two metrics. It reaches its maximum value when precision equals recall. All the mentioned metrics will be calculated for each class found in the confusion matrix and will be summarized in one table for each classification process.

(10) F1score=21/Precision+1/Recall.

The receiver operating characteristic (ROC) curve illustrates the performance of a classification model across all possible classification thresholds. This graphical representation plots the true positive rate (TPR) against the false positive rate (FPR) at various threshold levels. Where the TPR is the recall value and FPR is the ratio of FP over FP + TN. Lowering the classification threshold classifies more items as positive, thus increasing both FPs and TPs [1, 34, 35].

Area under the ROC curve (AUC) measures the entire two‐dimensional area underneath the entire ROC curve from (0, 0) to (1, 1). AUC ranges in value from 0 to 1. A model whose predictions are 100% wrong has an AUC of 0.0; one whose predictions are 100% correct has an AUC of 1.0. AUC is scale‐invariant, it measures how well predictions are ranked, rather than their absolute values and it is classification‐threshold‐invariant which means that it measures the quality of the model′s predictions irrespective of what classification threshold is chosen ([32]; [33]).

### 3.2. Results Using GB Method

Table 3 displays the classification result confusion matrix using the GB approach as seen in the Orang3 program. Out of the 1200 instances, 1188 cases from the Corona class had accurate predictions, compared with 1177 and 1150 cases for the normal and viral cases, respectively. The model misclassified the viral cases the most and classified them incorrectly as normal.

**Table 3:** Confusion matrix result using GB method.

Confusion matrix		Predicted classes		
		Corona Class 1	Normal Class 2	Viral Class 3
Actual classes	Corona Class 1	1188	2	10
	Normal Class 2	0	1177	23
	Viral Class 3	3	47	1150

The class‐wise performance evaluation of the GB classification model is presented in Table 4. The model achieved an overall accuracy of 97.64% on the testing dataset. Among the categories investigated, the Corona class showed the best performance, achieving a precision and recall of 99.0%, with only 12 misclassified samples. The normal class achieved a recall of 98.1% and an F1‐score of 97.0%, whereas the viral class obtained a precision of 97.2% and a recall of 95.8%. The slightly lower recall of the viral class was mainly attributed to confusion with normal cases, where 47 viral samples were incorrectly classified as normal. Despite these misclassifications, all classes achieved performance values above 95%, demonstrating the reliability of the proposed classification approach.

**Table 4:** Detailed evaluation results for GB method.

Class	TP	TN	FP	FN	Precision (%)	Recall (%)	F1‐score (%)
Corona	1188	2397	3	12	99.0	99.0	99.0
Normal	1177	2351	49	23	96.0	98.1	97.0
Viral	1150	2367	33	50	97.2	95.8	96.5
Average	—	—	—	—	97.4	97.6	97.5

### 3.3. Results Using RF Method

Table 5 displays the classification result confusion matrix using the RF approach as seen in the Orange3 program. Out of the 1200 instances, 1192 cases from the Corona and normal classes had accurate predictions, and 1183 cases for the viral disease type. The model misclassified the viral cases the most and classified them incorrectly as normal, almost the same as the GB model but RF had higher accuracy.

**Table 5:** Confusion matrix result using the RF method.

Confusion matrix		Predicted classes		
		Corona Class 1	Normal Class 2	Viral Class 3
Actual classes	Corona Class 1	1192	2	6
	Normal Class 2	0	1192	8
	Viral Class 3	2	15	1183

The class‐wise evaluation results of the RF classifier are presented in Table 6. The model achieved an overall accuracy of 99.08% on the testing dataset. The RF classifier correctly identified 1192 Corona cases, 1192 normal cases, and 1183 viral cases. The Corona class achieved the highest precision of 99.8% and a recall of 99.3%, indicating that the model effectively distinguished Corona samples with minimal false predictions. The normal class obtained a precision of 98.6% and a recall of 99.3%, while the viral class achieved a precision of 98.8% and a recall of 98.6%. The low number of FP and FN cases across all classes demonstrates the strong classification capability of the RF model. The F1‐scores were above 98% for all classes, confirming the balanced performance of the classifier in identifying the three categories.

**Table 6:** Detailed Evaluation results for RF method.

Class	TP	TN	FP	FN	Precision (%)	Recall/sensitivity (%)	F1‐score (%)
Corona	1192	2398	2	8	99.8	99.3	99.5
Normal	1192	2385	17	8	98.6	99.3	98.9
Viral	1183	2386	14	17	98.8	98.6	98.7
Average	—	—	—	—	99.1	99.1	99.0

### 3.4. Result Using AdaBoost Method

Table 7 displays the classification result confusion matrix using the AdaBoost approach as seen in the Orang3 program. In this model, all cases: the Corona, normal, and viral cases are all classified correctly with 100% accuracy. The AdaBoost model is the best in distinguishing between Corona, normal, and viral cases compared with RF and GB models.

**Table 7:** Confusion matrix result using AdaBoost method.

Confusion matrix		Predicted classes		
		Corona Class 1	Normal Class 2	Viral Class 3
Actual classes	Corona Class 1	1200	0	0
	Normal Class 2	0	1200	0
	Viral Class 3	0	0	1200

Based on Table 8, the AdaBoost classifier achieved perfect classification performance on the testing dataset, with an accuracy of 100%. All 3600 testing samples were correctly classified into their corresponding categories, including Corona, normal, and viral cases. The confusion matrix shows zero FP and FN cases for all classes. Consequently, the precision, recall, and F1‐score values reached 100% for each class, indicating complete separation among the three investigated categories within this dataset.

**Table 8:** Detailed evaluation results for RF method.

Class	TP	TN	FP	FN	Precision (%)	Recall/sensitivity (%)	F1‐score (%)
Corona	1200	2400	0	0	100.0	100.0	100.0
Normal	1200	2400	0	0	100.0	100.0	100.0
Viral	1200	2400	0	0	100.0	100.0	100.0
Average	—	—	—	—	100.0	100.0	100.0

Figure 7 summarizes the overall classification performance using the average values of AUC, CA, F1‐score, precision, and sensitivity for the three classes. AdaBoost achieved perfect performance, with an AUC of 1.000, CA of 100%, precision of 100%, recall (sensitivity) of 100%, and an F1‐score of 100%. RF also demonstrated excellent performance, achieving an accuracy of 99.1%, precision of 99.1%, recall of 98.6%, and an F1‐score of 99.1%. Similarly, GB achieved an accuracy of 97.6%, precision of 97.6%, recall of 96.5%, and an F1‐score of 99.7%. Although all three ensemble learning methods produced excellent classification performance, AdaBoost consistently outperformed RF and GB across all evaluation metrics.

**Figure 7:**
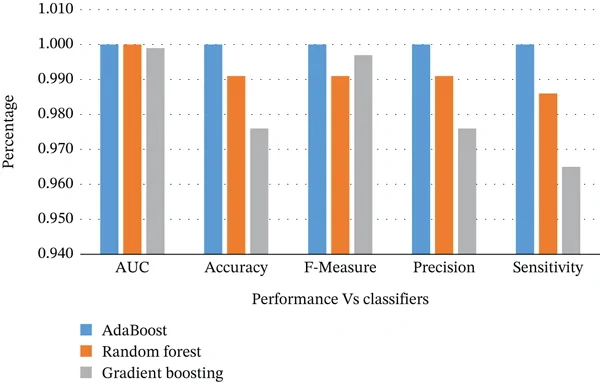
Comparison between the three methods.

### 3.5. ROC Curves

Figure 8 shows the ROC curve for all classifiers according to the Corona class, whereas Figures 9 and 10 show the ROC for the other two cases normal and viral. The ROC curves evaluate the trade‐off between the TPR (sensitivity) and the FPR across different classification thresholds. The curves for all three classifiers are concentrated near the upper‐left corner of the ROC space, indicating excellent discriminative ability. As summarized in Figure 7, all classifiers achieved AUC values close to 1.0 for the three classes, demonstrating that the extracted wavelet features provide highly discriminative information for separating COVID‐19, viral pneumonia, and normal CXR images. Among the evaluated classifiers, AdaBoost consistently achieved the highest AUC values, confirming its superior discrimination capability over RF and GB.

**Figure 8:**
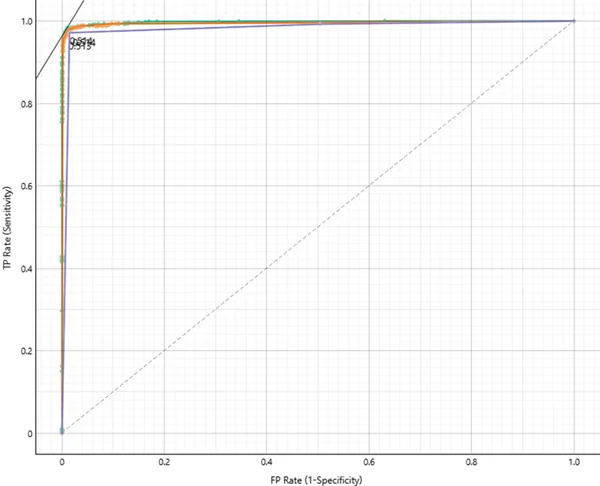
ROC curve for all classifiers according to Corona class.

**Figure 9:**
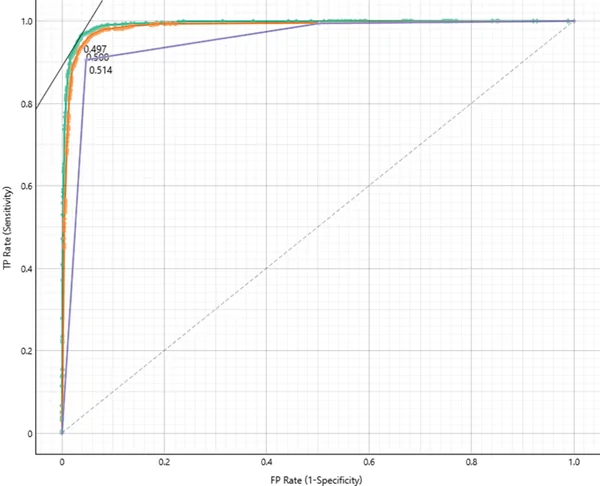
ROC curve for all classifiers according to normal class.

**Figure 10:**
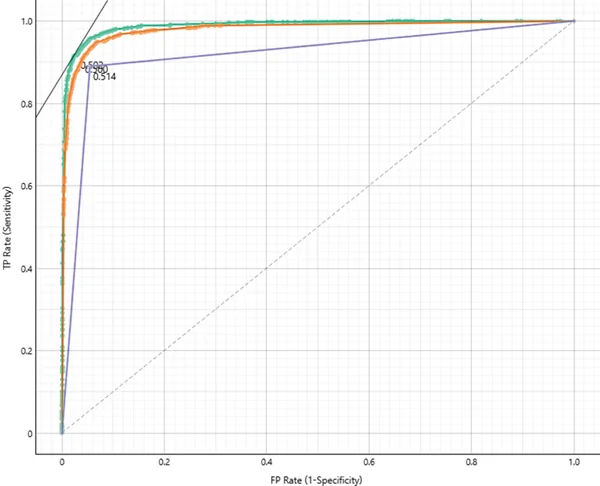
ROC curve for all classifiers according to viral class.

The near‐perfect ROC curves indicate that the proposed wavelet‐based feature representation effectively separates the three chest disease classes over a wide range of decision thresholds. This behavior suggests that the extracted texture and frequency‐domain characteristics contain highly discriminative information, allowing the classifiers to maintain high sensitivity while simultaneously minimizing FP classifications. Such performance is desirable in clinical screening applications, where reducing missed disease cases without increasing false alarms is essential.

### 3.6. Feature Importance

Table 9 presents the ranking of the five selected features according to information gain and gain ratio scores obtained using the rank widget in Orange3. The rank widget evaluates the relevance of each feature with respect to the target class by employing feature‐scoring measures such as information gain and gain ratio. Information gain quantifies the reduction in entropy achieved by a feature and reflects its discriminative ability for class separation. Gain ratio extends this measure by normalizing information gain with the intrinsic information of the attribute, thereby reducing the bias toward features with a large number of distinct values.

**Table 9:** Feature ranking based on gain values.

Feature	Information Gain	Gain ratio
Feature 5	0.871	0.351
Feature 1	0.831	0.338
Feature 4	0.804	0.319
Feature 3	0.783	0.306
Feature 2	0.697	0.270

As shown in Table 9, Feature 5 achieved the highest information gain (0.871) and gain ratio (0.351), indicating that it contributes the most to disease discrimination in CXR images. Feature 1 and Feature 4 ranked second and third, with information gain values of 0.831 and 0.804 and gain ratio values of 0.338 and 0.319, respectively. Feature 3 and Feature 2 exhibited comparatively lower scores, although they still provided meaningful discriminatory information. The consistent ranking obtained from both metrics suggests that Feature 5, Feature 1, and Feature 4 are the most informative features for distinguishing between different chest disease classes.

### 3.7. Comparison With Previous Work

Table 10 compares the performance of the proposed approach with several recent studies on CXR image classification. Most previous works employed deep learning and transfer learning models, including Inception‐V3, CheXNet, ResNet, DenseNet, Xception, and ensemble CNN architectures. These methods generally achieved high classification accuracies ranging from 84.95% to 99.9%, depending on the dataset size, disease classes, and classification task.

**Table 10:** Comparison with previous works in literature.

Author (year)	Number of Images/Dataset	Methodology	Results
Chhikara et al. (2021) [5]	Three chest radiography datasets: ~6500, 6871, and ~14000 images, respectively.	Transfer learning using Inception‐V3 with flattening, dropout, normalization, and dense layers for COVID‐19, normal, and viral pneumonia classification.	Accuracy: 97.7%, 84.95%, and 97.03% on the three datasets
Haritha et al. (2020) [6]	ChestXray14 dataset (1824 images)	CheXNet CNN model for binary classification (COVID‐19 vs. normal)	Accuracy: 99.9%
Chhikara et al. (2020) [7]	Standard x‐ray dataset from a private medical center (5866 images)	Deep CNN with transfer learning, image preprocessing (filtering, gamma correction, equalization, compression), compared with ResNet, Xception, Inception, and ImageNet‐based models. Binary classification (COVID‐19 vs. normal)	Outperformed conventional models according to accuracy (90.1%), precision (90.7%), recall (95.75%), and F1 score (93.15%)
Hashmi et al. (2020) [8]	The Guangzhou Women and Children′s Medical Center pneumonia dataset, they used 5836 chest x‐ray images	Ensemble weighted classifier combining ResNet18, Xception, InceptionV3, DenseNet121, and MobileNetV3 with transfer learning and data augmentation. Binary classification (normal vs. viral) pneumonia.	Combined classifier outperformed individual models with accuracy 98.43% and an AUC score of 99.76% on the unseen data.
Vogado et al. (2022) [36]	Chest x‐ray dataset: Normal: 236,350 and abnormal: 116,110.	Ensemble model composed of VGG‐16, ResNet50 and DenseNet121 architectures. Binary classification (normal vs. abnormal).	The classification accuracy is 87.54% and AUC score reached 87.21%.
Lenny et al. (2022) [11]	They used 5233 images for training and 624 for testing	Pretrained CNN models as feature extractors combined with many supervised classifiers for normal versus pneumonia classification.	Achieved high classification accuracy of 96% using InceptionV3.
Owida et al. (2021)[12]	Chest x‐ray images composed of 1200 normal and 1200 abnormal images.	Deep learning‐based diagnosis using Wavelet and MFCC feature extraction, SVM method is used for binary classification (normal vs. abnormal).	Wavelet features: 98.8% accuracy, MFCC features: ~97% accuracy.
Al‐Ghraibah et al. (2023) [1]	Chest x‐ray dataset 1200 images for two types of diseases.	Feature extraction using wavelet, SIFT, and MFCC, followed by SVM and ANN classification for COVID‐19 vs. viral pneumonia classes.	SIFT features: ~99% accuracy, wavelet features: 98.9% accuracy
Proposed work	Chest x‐ray dataset with 1200 images for each of the three classes.	Feature extraction using wavelet, classification into 3 classes: viral pneumonia, normal, and COIVD19.	AdaBoost with 100% accuracy, random forest with 99.1%, and gradient boosting with 97.6%

The proposed work achieved an accuracy of 100% using the AdaBoost classifier, outperforming most of the reported studies. RF and GB also demonstrated strong performance, achieving accuracies of 99.1% and 97.6%, respectively. Unlike many previous studies that relied on computationally intensive deep learning architectures, the proposed method utilizes wavelet‐based feature extraction combined with machine learning classifiers, resulting in a simpler and more interpretable framework. The obtained results are consistent with previous studies that highlighted the effectiveness of wavelet features for CXR analysis. For example, Owida et al. [12] and Al‐Ghraibah et al. [1] reported accuracies of 98.8% and 98.9%, respectively, using wavelet‐based approaches. The superior performance achieved in the present study suggests that the selected wavelet features effectively capture discriminative texture and structural information associated with COVID‐19, viral pneumonia, and normal CXR images.

It should be noted that direct comparison among studies should be interpreted with caution because different datasets, sample sizes, class distributions, preprocessing techniques, and evaluation protocols were used. Nevertheless, the comparison demonstrates that the proposed wavelet‐based framework is highly competitive with state‐of‐the‐art methods and provides excellent classification performance while maintaining lower computational complexity than many deep learning‐based approaches.

## 4. Conclusion

Using x‐ray images, the study classifies lung cancer, pneumonia, and viral pneumonia using machine learning approaches. High accuracy rates were attained by implementing algorithms using the Orang3 data mining tool. Patient care is enhanced, and radiologists′ work is decreased as a result of this integration. The study emphasizes how crucial reliable algorithms and cooperation between data scientists and medical experts are to future studies. Adaboost, GB, and RF techniques were utilized to develop classification models for the 1200 patients in each case′s dataset. Wavelet features were used to extract features, and they were fed to orange3 tool and the results accuracy of classifying the images using the proposed models were 99.5% on average. In the future, the number of diseases that are included in the classification process could be increased and be more general to help doctors choose the best course of treatment by accurately recognizing the type of disease from patient′s x‐ray chest image. The model could be merged with x‐ray machines in hospitals, and it can then produce results quickly once an image is taken.

## Author Contributions

**Amani Al-Ghraibah:** conceptualization, methodology, formal analysis, writing – original draft. **Areen Arabiat:** data curation, software, validation, writing – review and editing. **Muneera Altayeb:** supervision, project administration, and funding acquisition.

## Funding

No funding was received for this manuscript.

## Conflicts of Interest

The authors declare no conflicts of interest.

## Data Availability

The data that support the findings of this study are available from the corresponding author upon reasonable request.
